# Sanitation Practices during Early Phases of COVID-19 Lockdown in Peri-Urban Communities in Tamil Nadu, India

**DOI:** 10.4269/ajtmh.20-0830

**Published:** 2020-09-29

**Authors:** Sania Ashraf, Jinyi Kuang, Upasak Das, Cristina Bicchieri

**Affiliations:** 1 Center for Social Norms and Behavioral Dynamics, University of Pennsylvania, Philadelphia, Pennsylvania;; 2 Global Development Institute, University of Manchester, Manchester, United Kingdom

## Abstract

In countries without adequate access to improved sanitation, government-imposed restrictions during the COVID-19 pandemic can impact toilet usage. In India, where millions have recently transitioned to using a toilet, pandemic-related barriers to use might increase open defecation practices. We assessed changes in reported defecation practices in peri-urban communities in Tamil Nadu. Field assistants conducted phone surveys in 26 communities in two districts from May 20, 2020 to May 25, 2020. They asked respondents about their access to a toilet, whether they or a family member left their house to defecate in the past week, and whether specific practices had changed since the lockdown. Among 2,044 respondents, 60% had access to a private toilet, 11% to a public or community toilet, whereas 29% lacked access to any toilet facility. In our study, 92% of the respondents did not change their defecation behaviors in the 2 months following the pandemic-related lockdown. About a third (27%) reported that they or a family member left their house daily to defecate amid lockdown measures. A majority of those with private toilets (91%) or with public toilets (69%) continued using them. Respondents with private toilet access were more likely to report an increased frequency of handwashing with soap (prevalence ratio [PR]: 1.78, 95% CI: 1.04–3.05) since the lockdown. The lack of private toilets contributes to the need to leave the house amid a lockdown. Maintaining shared toilets require disinfection protocols and behavioral precautions to limit the risk of fomite transmission. Robust urban COVID-19 control strategies should include enhanced sanitation facility management and safe usage messaging.

## INTRODUCTION

Respiratory and hand hygiene focused behavior change is critical to limit the spread of SARS-CoV-2, the etiologic agent of the ongoing COVID-19 pandemic.^1,2^ The primary transmission route for COVID-19 is respiratory droplets and fomites from infected persons, which led to the promotion of preventative measures such as face masks, regular handwashing with soap, and maintaining a minimum distance in public areas.^2^ However, emerging evidence highlights the presence of detectable virus particles in wastewater, and feces of infected cases COVID-19.^3–5^ This led to calls to evaluate the possibility of fecal–oral transmission in countries without safely managed sanitation^6–8^ and initiatives to assess wastewater for environmental surveillance to detect ongoing transmission.^9^

In India’s context, where more than 74 million are homeless or live in slums, controlling this infectious disease requires targeted policies to provide access to sanitation facilities, soap, and water in addition to social distancing.^10,11^ India is also unique because it has recently transitioned millions who previously defecated in the open to using a toilet.^12^ A decade ago in 2011, the national census highlighted that 49.8% of the population in India reported defecating in the open.^13^ Since then, toilet coverage and toilet usage have increased following concerted efforts by the government and NGOs.^13,14^ In 2017 the WHO/UNICEF Joint Monitoring Programme (JMP) estimated that 60% of the population had access to safely managed and basic sanitation.^13^ However, several studies have highlighted the prevalence of inconsistent toilet use, and persistent open defecation practices continue to be a challenge.^15,16^ Despite having access to private toilets, many household members do not exclusively use them.^15^ Given the limitations imposed by a lockdown, more household members are expected to strictly stay at home. It is important to examine whether this led to changes in the use of private toilets, that is, if more household members use the toilet, or if it potentially led to open defecation practices by some members. In the event, that there are elderly family members, higher perceived risk of using the same toilet among household members may also impact toilet use. Related concerns may also increase hygiene-related behaviors such as frequency of toilet cleaning and handwashing with soap. In addition, a considerable proportion also relies on community or pay-per-use public toilets especially in urban and peri-urban areas.^17–19^ Shared sanitation facilities are prone to unhygienic conditions due to poor maintenance.^18,20,21^ The COVID-19 crisis may intensify these specific challenges because use by multiple households can increase the risk of contact and formite exposures.^1^ As social distancing measures restrict movement, it may limit access to public or communal toilets. It is therefore important to understand whether such barriers have increased open defecation practices to avoid potential perceived risks of using shared toilet facilities.^22^

In response to the COVID-19 pandemic, on March 24, 2020, India imposed a nationwide lockdown, restricting the movement of 1.3 billion people.^11^ In Tamil Nadu, national lockdown measures allowed movement within districts between 6 am and 7 pm to complete essential duties. We aimed to assess whether in communities with inadequate access to sanitation, daily defecation behaviors and hygiene behaviors changed in response to the pandemic.

We conducted a cross-sectional survey among households in peri-urban communities to 1) assess whether respondents stepped out of their house for defecation purposes during the lockdown; 2) what changes, if any, occurred in toilet use during the lockdown?; 3) what changes, if any, occurred in reported toilet cleaning and handwashing with soap during the lockdown?

## METHODS

### Study setting and design.

We conducted a cross-sectional study among participants conveniently sampled from households enrolled in an ongoing cluster-randomized trial in two districts, Pudukkottai and Karur, in Tamil Nadu. Within these districts, we had access to peri-urban wards that were enrolled as a part of a larger randomized controlled trial called the Longitudinal Evaluation of Network and Norms Study (LENNS).^23^ These wards are close to urban cities and include many migrant workers who live below the poverty line and have poor sanitation conditions.^24^ At the time of this survey, there were at least 144,941 confirmed COVID-19 cases in India, with 4,171 death cases, and 17,082 confirmed cases in Tamil Nadu, with 118 deaths.^25^ To the best of our knowledge, there were no active cases reported in these districts at the time of the survey. We conducted this study during a nationwide COVID-19–related lockdown, which allowed residents to leave their houses for essential activities only during certain hours of the day. Notably, this lockdown was among the strictest in the world according to a severity measure developed by the Blavatnik School of Government at Oxford University.^26^ The lockdown led to a national-level migration out of major cities and an increase in unemployment rates by more than 23.5% from March to April 2020.^27^

### Selection of participants.

For logistical considerations, we used a convenient sample from a sampling frame of 2,657 phone numbers from households in randomly selected peri-urban wards across the two districts. The list of phone numbers was generated through an in-person survey in February during the enrollment of eligible households for the LENNS trial. The phone numbers were from individuals who volunteered to participate in group texting activities as a part of the LENNS behavior change strategy. Only one phone number per enrolled household was included. Because of restriction of movement imposed by the government of India, in-person surveys were not feasible. Accessing the wards enrolled in an existing study allowed us to leverage established rapport within the communities and engage them in this phone survey. In particular, before the lockdown, we had audio announcements in all the wards and community engagement meetings in a subset of them introducing the study and its objectives to the local resident and town panchayat officials.

### Data collection.

Field-workers conducted phone surveys from May 20, 2020 to May 25, 2020 across the two districts. We designed a structured questionnaire to collect data on toilet use behaviors, sociodemographic variables, and changes in behavior to mitigate potential risks during the pandemic. Field-workers also asked respondents about their and their family members’ toilet use behavior, and whether there were any changes to their usual toilet usage and cleaning practices because of the lockdown.

The survey was translated and tested by bilingual researchers to ensure verbal comprehension and ease of implementation. Ten field-workers were trained remotely on the instrument and included phone-based techniques, relevant answers to COVID-19–related questions, and clarifications they might receive during the call. All calls were conducted in the local language Tamil and lasted between 20 and 25 minutes. The calls were made between 10 am and 5 pm. A number was attempted at least twice in the same day, before being considered unreachable. The target respondent was whoever answered the phone, if they were an adult residing in that ward and consented to participating in the survey. Data were collected using personal handheld devices and used a digital data collection form. A project manager supervised the team daily to ensure data consistency and completeness of data entry. Field assistants were employed by Swasti, a local nongovernmental organization in partnership with the University of Pennsylvania.

### Outcome measurement.

We informed the participants that the purpose of the survey was to understand how the COVID-19 pandemic is impacting their health and well-being. We asked respondents “Do you have access to a toilet?” Responses included private toilet, shared private toilet, community, or public toilet. Participants’ perceived risk of personally contracting coronavirus was categorized as no risk, low, medium, and high risk. To understand defecation practices, we gathered information on the reasons the respondent or a family member stepped out of the household in the past week in an open-ended question; responses were coded to include food shopping, medicine shopping, open defecation, public/community toilet, exercise, and to meet family and friends. We used this variable “to go out for open defecation” or “use a toilet by any family member” as the main outcome of interest to indicate risk of exposure to COVID-19. We also collected data on their open defecation practice in the past 2 days. To understand the impact on their daily sanitation behaviors, we asked them if, compared with before the lockdown, there were any changes in their defecation routine, handwashing with soap, or toilet cleaning frequency. We also asked them “In the past 7 days, have you or anyone in your household eaten less or skipped meals because you did not have enough to eat?” as a measure of food insecurity. We included this as a proxy for socioeconomic status in our analyses because of the sensitivity of questions about household income or assets on phone surveys. Some of our items were influenced or adapted from other COVID-19–related surveys to allow comparison across studies.^28^

### Data analysis.

We calculated descriptive statistics of the variables of interest and assessed differences across sociodemographic groups such as age, gender, and education levels, using Pearson’s chi-square test to test the equality of proportions. We calculated the prevalence ratio using log binomial models to evaluate the association between individual and household characteristics with stepping out of the household for open defecation or to use a toilet.^29^ We built multivariable models using hierarchical conceptual frameworks and included potential confounders that were associated with the outcome of interest at *P* < 0.1 level in bivariate analyses.^30^ To account for clustering in the ward level, we used general estimated equations to calculate these relative prevalence ratios and 95% CIs. Analyses were performed using Stata 14.0 (Stata Corp LP, College Station, TX).

### Ethical consideration.

The ongoing trial and the amendment to conduct this survey was reviewed and approved by the ethical board at the University of Pennsylvania (833854). All respondents were asked for verbal informed consent on the phone before starting the survey.

### Role of the funding source.

The sponsor of the study, the Bill & Melinda Gates Foundation, had no role in the study design, data collection, analysis, or interpretation.

## RESULTS

Of 2,657 dialed numbers, a total of 2,330 respondents (88%) participated in the phone survey. In our analysis, we included a total of 2,044 respondents who completed the full survey (female = 47%). The average age is 44 (SD = 14) years, ranging from 18 to 90 years. Among sampled respondents, 34% had completed high school education or above. On average, there were four people living in the same household at the time of the survey (SD = 1.5). The majority reported the drinking water source was located within the household unit (62%), and the gas stove was the primary cooking fuel (88%) (Table 1).

**Table 1:** Sociodemographic characteristics of study population, May 2020, Tamil Nadu, India

*N* = 2,044	Pudukkottai (*n* = 722), *n* (%)	Karur (*n* = 1,322), *n* (%)	Total, *n* (%)
Age (years), mean (SD)	45 (14)	44 (15)	44 (14)
Female	346 (48)	608 (46)	954 (47)
No. of household members	4.2 (1.5)	3.8 (1.5)	3.9 (1.5)
Education			
None	140 (19)	223 (17)	363 (19)
Primary (1–5 yr)	199 (28)	225 (17)	424 (21)
Secondary (6–10 yr)	174 (24)	393 (30)	567 (28)
High school (11–12 yr)	137 (19)	246 (19)	383 (19)
University (12 yr+)	72 (10)	235 (18)	307 (15)
Toilet access			
None	231 (32)	358 (27)	589 (29)
Private toilet	475 (66)	760 (57)	1,235 (60)
Community toilet	6 (0.8)	6 (0.5)	12 (0.6)
Public toilet	10 (1.4)	198 (15)	208 (10)
Water source			
In their own house	156 (22)	875 (66)	1,264 (62)
In own plot/yard	389 (54)	143 (11)	299 (15)
Elsewhere	177 (25)	304 (23)	481 (24)
Fuel			
LPH/gas stove	566 (78)	1,213 (92)	1,779 (87)
Wood	142 (20)	85 (6.4)	227 (11)
Kerosene	10 (1.4)	10 (0.8)	20 (0.9)
Biogas	1 (0.1)	3 (0.2)	4 (0.2)

Data are *n* (%). LPH = low pressure heater.

### Sanitation practices.

Among 2,044 respondents, 1,235 (60%) had access to a private toilet. About a third (*n* = 589, 29%) of our respondents did not have access to any toilet facility. The overall coverage of public toilets was low, where only 208 (10%) reported that they had access to one (Table 1). We combined proportions of community and public toilets in further analyses because of the limited number of community toilets (*n* = 12) in our sample (0.6%). Both are considered shared sanitation facilities. Community toilets are used by a defined group of local residents as their main toilet facility. These are commonly managed by the residents or local authorities. Public toilets are sanitation facilities that are open to all, located in high-traffic public areas.^31^

Six hundred forty-seven (32%) respondents reported open defecation in the past 2 days. This was reported by 84% of those who did not have access to any toilet facilities, 45% with access to public/community toilets, and 4.4% of those with private toilet facilities. When asked specifically about leaving the house to defecate in the open (OD), 403 (20%) reported either they or a family member did so in the past week. This was reported by 58% of respondents who did not have access to any toilet facility and 16% of respondents with access to public/community toilet. Only 2% of those with private toilet needed to leave the house for defecation purposes. We noted the discrepancy between the OD prevalence in the past 2 days (32%) and whether they or a family member left the house to OD in the past 7 days (20%). One explanation of this is because the latter was asked as an open-ended question, where possible responses were not read out. This may have led to an underestimation of this estimate.

In our sample, although men were less likely to report open defecation in the past 2 days than women (28% versus 36%), the difference was not significant once we accounted for the access to toilet facilities. Other than that, there were no meaningful differences in sanitation practices by gender.

Most respondents reported that their defecation practices did not change (*n* = 1,886, 92%) since the lockdown. Among respondents with access to a private toilet (*n* = 1,235), the majority continued using the toilet (91%), or reported that they (6.4%) or a family member started using them (7.3%) as a result of the pandemic. Of those without access to a toilet (*n* = 589), 490 (83%) continued defecating in the open, whereas another 28 (5%) started to defecate in the open (OD) during the lockdown.

Among those with access to public toilets or community toilets (*n* = 220), the majority (69%) said they were continuing using it, whereas some (13%) respondents said they started using it during the lockdown. Thirty-three (15%) respondents with access to these shared toilers reported continuing to defecate in the open. We found evidence of misreporting, where some respondents (8.8%) said they continued using a community/public toilet during this time, despite previously stating that they did not have access to a toilet (Table 2). In general, we did not find evidence that respondents were avoiding public toilets during the lockdown.

**Table 2:** Sanitation and hygiene-related behavior during a COVID-19–related lockdown by gender and toilet access, Tamil Nadu, India, May 2020

Behavior of interest	Total (*N* = 2,044), *n* (%)	Gender		Toilet access		
		Female (*N* = 954), *n* (%)	Male (*N* = 1,090), *n* (%)	No toilet (*N* = 589), *n* %)	Private toilet (*N* = 1,235), *n* (%)	Public or community toilet (*N* = 220), *n* (%)
Reported open defecation in the past 2 days	647 (32)	342 (36)*	305 (28)	495 (84)	54 (4.4)	98 (45)
Respondent or a family member left the house for open defecation in the past 7 days	403 (20)†	235 (25)	168 (16)	344 (58)	25 (2.0)	34 (16)
Respondent or a family member left the house to use a toilet in the past 7 days	157 (7.7)	89 (9.3)	68 (6.2)	33 (5.6)	20 (1.6)	104 (47)

Changes in behaviors						
Respondent continued using a private toilet	1,130 (56)	457 (48)	673 (62)	7 (1.2)‡	1,117 (91)	6 (2.7)
Continued open defecation	542 (27)	294 (30)	248 (23)	490 (83)	19 (1.5)	33 (15)
Continued using a public/community toilet	214 (11)	84 (7.7)	130 (14)	52 (8.8)§	10 (0.8)	152 (69)
Started using a private toilet	80 (3.9)	60 (2.6)	30 (3.1)	0	80 (6.5)	0
Started using a public toilet	48 (2.4)	15 (1.4)	33 (3.5)	12 (2.0)	8 (0.7)	28 (13)
Started open defecation	30 (1.5)	5 (0.5)	22 (2.0)	28 (4.8)	1 (0.1)	1 (0.5)
Increased frequency of cleaning toilets	858 (42)	373 (39)	485 (45)	0	858 (70)	0
Increased washing hands with soap and water	1,723 (85)	806 (85)	917 (84)	461 (78)	1,074 (87)	188 (86)

Data are *n* (%).

* Although men were less likely to report open defecation in the past 2 days, the difference was not significant once the access to toilet facilities was accounted for.

† Asked as an open-ended question, which may have led to fewer relevant responses.

‡ May indicate use of a private toilet owned by someone else.

§ Indicates misclassified respondents, who had access to public toilets despite reporting that they did not have access to any toilet.

Respondents who owned a private toilet (PR = 0.01, 95% CI: 0.01–0.04) or had access to a community or public toilet (PR = 0.14, 95% CI: 0.04–0.50) were less likely to go out of their households for open defecation (Table 3). As expected, going out to use a toilet was strongly associated with having access to a public or community toilet (PR = 14.7, 95% CI: 4.9–43.6) (Table 3).

**Table 3:** Multivariable analysis of factors associated with leaving the house for defecation purposes in the past week in Tamil Nadu, 2020

Characteristic	Went out for od in the past week (*n* = 403), *n* (%)	Prevalence ratios, PR (95% CI)	Adjusted prevalence ratios aPR* (95% CI)	Went out to use a toilet in the past week (*n* = 157), *n* (%)	PR (95% CI)	aPR* (95% CI)
Access to toilet						
None	344 (86)	Ref	Ref	33 (5.6)	Ref	Ref
Private	25 (6.2)	0.02 (0.01-0.39)	0.014 (0.01-0.04)	20 (1.6)	0.28 (0.09-0.86)	0.25 (0.10-0.79)
Community/Public	34 (8.4)	0.13 (0.37-0.46)	0.14 (0.04-0.50)	104 (47.3)	15.1 (4.93-46.32)	14.6 (5.07-42.3)

* Multivariable model adjusts for age, gender, reported food insecurity, respondent’s education level, and clustering at the ward level.

### Hygiene-related practices.

Most of the toilet owners (70%) reported an increase in the frequency of cleaning their toilets since the lockdown. A majority of the respondents (85%) said their frequency of handwashing with soap and water increased compared with before the lockdown. Among them, a majority (62%) had access to a private toilet. Compared with those without access to a toilet, respondents of the same age, gender, and education were more likely to report they increased the frequency of handwashing since the lockdown if they had a private toilet (adjusted prevalence ratio (aPR): 1.78, 95% CI: 1.04–3.05) (Table 4).

**Table 4:** Association of toilet access with reported increased handwashing since the lockdown in Tamil Nadu, 2020

	Increased washing hands with soap and water since lockdown, *n* (%)	PR (95% CI)	aPR (95% CI)*
*N* = 2,044	1,723 (84)	–	–
No access	461 (78)	Ref	Ref
Private toilet	1,074 (87)	1.85 (1.06–3.25)	1.78 (1.04–3.05)
Community/public toilet	188 (86)	1.63 (0.63–4.2)	1.61 (0.64–4.06)

* Adjusted for age, gender, and respondent’s education level.

## DISCUSSION

In peri-urban Tamil Nadu, not having access to a private toilet necessitated a considerable proportion of respondents to leave their house daily for defecation purposes amid a national lockdown. In communities with active transmission, there is a need to add adequate prevention measures such as using face masks and social distancing because people might meet others on the way or near the site. COVID-19 can manifest as severe illness, especially in the older age-groups. Concerningly, an estimated 2–10% of confirmed COVID-19 cases have diarrhea which increases the need to use convenient toilets.^32^ This pandemic context heightens the convenience of owning a private toilet and adds to the burden of caring for a patient with a highly contagious infection in the absence of proper sanitation facilities.^33^ In the context of India, a lot of the associated care-giving roles are likely to fall on women.^34–36^

At the time of this study, we did not find evidence that people changed their sanitation behavior in response to the pandemic. For those without access to a private toilet, open defecation practices and use of public toilets were considered necessary activities amid mass communication to stay indoors and maintain social distancing. Despite low access to public toilets in our study communities, a high proportion of respondents continued using them during the pandemic. In a public facility used daily by community members, maintaining proper management during a pandemic requires clear guidance, funding, and community engagement. This includes behavioral measures such as maintaining social distance, wearing masks, hand hygiene, and safe waste disposal while using the facility. The WHO guidelines specify disinfection of sanitation facilities that encourages individual and separate toilet facilities for exposed or ill persons.^37^ These recommendations emphasize disinfection at least twice per day by trained cleaners.^22^ In the context of public toilets in low-income communities, this requires implementation of standard operating procedures for regular disinfection by trained sanitation workers who are equipped with proper personal protective equipment (PPE).^38^ This can be challenging, given known shortages in PPE, because of the high demand across a range of frontline workers and health officials.^39^ Public sanitation facilities should also have adequate soap and water for the users and cleaning staff to practice proper hand hygiene to minimize transmission risks.^33^ In the absence of such a commitment, these toilets might transform into SARS-CoV-2 transmission hotspots for their users. These concerns are consistent with those emerging from the active COVID-19 transmission in Dharavi, Mumbai, the largest slum in India.^40^

Few respondents also started using a public toilet during the lockdown (2.4%), suggesting uninvestigated shifts in toilet use. Further research is required to understand whether these respondents shifted from open defecation practices or using toilets owned by other households in response to the pandemic. Because of limitations of a phone survey and assumed heightened sensitivity to new COVID-19–related information, we did not probe whether respondents perceived usage of public toilets as a health hazard. Additional data collected through this survey suggested that the perceived risk of contracting COVID-19 was low or none among the respondents.^41^ This may be a reaction to the relative low number of cases in those specific districts at the time of the survey. Given the recent sharp increase in COVID-19 cases in Tamil Nadu recently, it is a valid concern if exposed, asymptomatic, or ill persons continue to use shared sanitation facilities.

In our study, the prevalence of open defecation, despite access to toilets (private and public), reflects communities transitioning to exclusive toilet use. Although less than 2% reported that they started open defecation since the lockdown, it adds to the 27% who reported continuing to defecate in the open during the pandemic. Given the presence of open defecation behavior even among toilet owners, behavioral slippage or reversion to previous open defecation practices is possible, especially among new toilet users.^42,43^ Sustaining toilet use requires behavioral maintenance strategies, including adequately maintained facilities that support consistent use. Although we did not assess these factors in our study, known barriers such as increases in perceived risks from waiting in queues to use the shared toilets or using contaminated toilet facilities could inadvertently lead to increases in inconsistent toilet use and open defecation. This will weaken the results of long-standing national sanitation focused behavior change campaigns, the latest of which was the Swachh Bharat Mission launched in 2014 to end open defecation in India. This program accelerated toilet coverage using monetary subsidies and used careful behavior change strategies to promote exclusive toilet usage.^44,45^ To ensure continued toilet use during the pandemic, careful messaging is needed to engage shared toilet users and reassure them that measures are being taken to minimize risks of COVID-19 transmission.

Reporting of increased handwashing with soap compared with before the lockdown is indicative of the respondents’ knowledge of the related benefits of this widely promoted preventative measure. In particular, we found that respondents with access to a private toilet were more likely to report that they increased handwashing with soap since the lockdown. Having access to a toilet may increase habitual handwashing with soap by providing a stable environment for handwashing after fecal contact, or in general after possible fomite exposure during the pandemic. This is consistent with findings that washing hands with soap was more common when soap and water are together in a convenient place.^46^ Handwashing with soap is a key preventative measure against SARS-CoV-2 transmission.^2^ Increasing access to equipped handwashing stations is a key public health priority during a pandemic. For households without access to a private toilet, strategies to encourage them to designate equipped handwashing stations at other easily accessible locations such as the kitchen, the courtyard, or near their place of worship should be explored.^47^ Further research is required to confirm the positive spillover effect of toilet access and increased frequency of handwashing with soap during the pandemic in resource-poor settings.

This study has several limitations. Phone surveys are time constrained and may not allow enumerators to adequately assess the participant’s level of understanding or engagement with the questions asked.^48^ For example, we found evidence that some respondents, when asked about toilet access, assumed reference to a private toilet, leading to possible underestimation of access to public or community toilets. Next, this study is nested in a behavior change study focused on increasing exclusive toilet use. At the time of data collection, the study did not proceed with considerable behavior change activities and was paused because of COVID-19–related restrictions. It is possible that the respondents associated the survey with the overall project and underreported open defecation because of a social desirability bias. This is unlikely because we clarified that the aim of the survey was to understand how the pandemic and lockdown impacted their daily behaviors and well-being. Moreover, this is also an observational study and is therefore subject to unmeasured confounding. However, given that the primary outcome was based on whether they went out of the household for a specific activity during a serious pandemic with widespread instructions to stay at home, the responses are likely to reflect habits or a necessity. In addition, we controlled for a range of potential confounding factors to minimize bias. Next, self-reported handwashing is known to be subject to social desirability bias, especially in a pandemic setting, where it is paired with social expectations for the common good.^49^ Last, although these findings provide us with relevant insights about sanitation-related behaviors of concern, they were collected when no known COVID-19 cases were reported in these districts. Toilet use and related behaviors are expected to change as the pandemic intensifies and further restrictions are imposed. Indeed, it is possible that an assessment longer into the lockdown would have better captured changes in sanitation practices and toilet usage in these communities. Further research should assess the impact on toilet use and sanitation practices in communities with inadequate access to improve sanitation.

## CONCLUSION

In countries with prevalent open defecation and a high reliance on shared toilets for those without private toilets, careful messaging is required to sustain safe sanitation facilities during this pandemic. Any robust inclusive COVID-19 control strategy in peri-urban India and in low-income urban communities should include enhanced sanitation facility management that includes adequate handwashing facilities and widespread messages to promote safe usage. The prime sanitation-focused public health agenda in India in the past decade should be adequately reflected in the public policies implemented during this crucial crisis.

## References

[1] WHO, 2020 Water, Sanitation, Hygiene and Waste Management for the COVID-19 Virus: Interim Guidance April 2020. Geneva, Switzerland: World Health Organization.

[2] World Health Organization, 2020 Coronavirus Disease (COVID-19) Advice for the Public. Available at: https://www.who.int/emergencies/diseases/novel-coronavirus-2019/advice-for-public.

[3] Wölfel R 2020 Virological assessment of hospitalized patients with COVID-2019. Nature 581: 465–469. 32235945 10.1038/s41586-020-2196-x

[4] Zhang W 2020 Molecular and serological investigation of 2019-nCoV infected patients: implication of multiple shedding routes. Emerg Microbes Infect 9: 386–389. 32065057 10.1080/22221751.2020.1729071 PMC7048229

[5] Gu J Han B Wang J, 2020 COVID-19: gastrointestinal manifestations and potential fecal–oral transmission. Gastroenterology 158: 1518–1519. 32142785 10.1053/j.gastro.2020.02.054 PMC7130192

[6] Tian Y Rong L Nian W He Y, 2020 Review article: gastrointestinal features in COVID-19 and the possibility of faecal transmission. Aliment Pharmacol Ther 51: 843–851. 32222988 10.1111/apt.15731 PMC7161803

[7] Odih EE Afolayan AO Akintayo I Okeke IN, 2020 Could water and sanitation shortfalls exacerbate SARS-CoV-2 transmission risks? Am J Trop Med Hyg 103: 554–557. 32524953 10.4269/ajtmh.20-0462 PMC7410451

[8] Heller L Mota CR Greco DB, 2020 COVID-19 faecal-oral transmission: are we asking the right questions? Sci Total Environ 729: 138919. 32353720 10.1016/j.scitotenv.2020.138919 PMC7182518

[9] Bivins A 2020 Wastewater-based epidemiology: global collaborative to maximize contributions in the fight against COVID-19. Environ Sci Technol 54: 7754–7757. 32530639 10.1021/acs.est.0c02388

[10] Sur P Mitra E, 2020 Social distancing is a privilege of the middle class. For India’s slum dwellers, it will be impossible. CNN. Available at: https://www.cnn.com/2020/03/30/india/india-coronavirus-social-distancing-intl-hnk/index.html. Accessed March 30, 2020.

[11] The Lancet, 2020 India under COVID-19 lockdown. Lancet 395: 1315. 32334687 10.1016/S0140-6736(20)30938-7 PMC7180023

[12] Government of India, 2014 Guidelines for Swachh Bharat Mission (Gramin): Ministry of Drinking Water and Sanitation. Available at: http://www.and.nic.in/rdpri/downloads/guidelines_Swachh_Bharat_Mission_Gramin.pdf. Accessed September 7, 2018.

[13] WHO/UNICEF, 2017 WHO|Progress on Drinking Water, Sanitation and Hygiene. Geneva, Switzerland: World Health Organization Available at: http://www.who.int/water_sanitation_health/publications/jmp-2017/en/. Accessed August 22, 2017.

[14] Grameen SB, 2020 SBM-G Phase-II Launched. Available at: https://sbmgramin.wordpress.com/2020/03/06/sbm-g-phase-ii-launched/.

[15] Barnard S Routray P Majorin F Peletz R Boisson S Sinha A Clasen T, 2013 Impact of Indian total sanitation campaign on latrine coverage and use: a cross-sectional study in Orissa three years following programme implementation. PLoS One 8: e71438. 23990955 10.1371/journal.pone.0071438 PMC3749227

[16] Coffey D Gupta A Hathi P Khurana N, 2014 Revealed preference for open defecation: evidence from a new survey in rural north India. Squat Rep 6: 1–42.

[17] Ghosh A Cairncross S, 2014 The uneven progress of sanitation in India. J Water Sanit Hyg Dev 4: 15.

[18] Biran A Jenkins MW Dabrase P Bhagwat I, 2011 Patterns and determinants of communal latrine usage in urban poverty pockets in Bhopal, India. Trop Med Int Health 16: 854–862. 21414114 10.1111/j.1365-3156.2011.02764.x

[19] WSP, 2016 Community Slum Sanitation in India. Available at: https://www.wsp.org/sites/wsp.org/files/publications/Community Slum Sanitation in India.pdf.

[20] Günther I Niwagaba BC Lüthi C Horst A Mosler H-J Tumwebaze KI, 2012 When is shared sanitation improved sanitation? The correlation between number of users and toilet hygiene. Res Policy Eidgen{ö}ssische Tech Hochschule Z{ü}rich [ETHZ]. Zurich, Switzerland: Research for Policy.

[21] Heijnen M Cumming O Peletz R Chan GKS Brown J Baker K Clasen T, 2014 Shared sanitation versus individual household latrines: a systematic review of health outcomes. PLoS One 9: e93300. 24743336 10.1371/journal.pone.0093300 PMC3990518

[22] Caruso BA Freeman MC, 2020 Shared sanitation and the spread of COVID-19: risks and next steps. Lancet Planet Health 4: e173. 10.1016/S2542-5196(20)30086-3. 32442489 PMC7237180

[23] Ashraf S Bicchieri C Delea MG Das U Chauhan K Kuang J Shpenev A McNally PK Thulin E, 2020 Design and rationale of the Longitudinal Evaluation of Norms and Networks Study (LENNS): a cluster-randomized trial assessing the impact of a norms-centric intervention on exclusive toilet use and maintenance in peri-urban communities of Tamil Nadu. medRxiv, 10.1101/2020.06.26.20140830.

[24] International Institute of Population Sciences (IIPS), 2016 National Family Health Survey (NFHS-4). Available at: http://rchiips.org/nfhs/.

[25] Government of India, Ministry of Health and Welfare, 2020 Available at: https://www.mohfw.gov.in/. Accessed February 7, 2020.

[26] University of Oxford, 2020 Coronavirus Government Response Tracker. Available at: https://www.bsg.ox.ac.uk/research/research-projects/coronavirus-government-response-tracker. Accessed September 7, 2020.

[27] CMIE, 2020 The Jobs Bloodbath of April 2020. Available at: https://www.cmie.com/kommon/bin/sr.php?kall=warticle&dt=2020-05-05 08:22:21&msec=776. Accessed September 7, 2020.

[28] Innovations for Poverty Action, 2020 RECOVR Questionnaire Repository. Available at: poverty-action.org/recovr/questionnaire-repository.

[29] Barros AJD Hirakata VN, 2003 Alternatives for logistic regression in cross-sectional studies: an empirical comparison of models that directly estimate the prevalence ratio. BMC Med Res Methodol 3: 1–13. 14567763 10.1186/1471-2288-3-21 PMC521200

[30] Victora CG Huttly SR Fuchs SC Olinto MTA, 1997 The role of conceptual frameworks in epidemiological analysis: a hierarchical approach. Int J Epidemiol 26: 224–227. 9126524 10.1093/ije/26.1.224

[31] WSP, 2011 Community Sanitary Complexes In Rural Areas. New Delhi, India Available at: https://swachhbharatmission.gov.in/sbmcms/writereaddata/images/pdf/technical-notes-manuals/Community-Sanitary-Complex.pdf.

[32] Chen N 2020 Epidemiological and clinical characteristics of 99 cases of 2019 novel coronavirus pneumonia in Wuhan, China: a descriptive study. Lancet 395: P507–P513. 10.1016/S0140-6736(20)30211-7 PMC7135076 32007143

[33] World Health Organization, 2020 Home Care for Patients with COVID-19 Presenting with Mild Symptoms and Management of Their Contacts. Geneva, Switzerland: WHO.

[34] Sahoo KC Hulland KRS Caruso BA Swain R Freeman MC Panigrahi P Dreibelbis R, 2015 Sanitation-related psychosocial stress: a grounded theory study of women across the life-course in Odisha, India. Soc Sci Med 139: 80–89. 26164119 10.1016/j.socscimed.2015.06.031

[35] Wijk-Sijbesma C, 2016 Gender in Water Resources Management, Water Supply and Sanitation: Roles and Realities Revisited. The Hague, The Netherlands: Water Policy.

[36] Li X 2020 Risk factors for severity and mortality in adult COVID-19 inpatients in Wuhan. J Allergy Clin Immunol 146: 110–118. 32294485 10.1016/j.jaci.2020.04.006 PMC7152876

[37] World Health Organization, 2020 Water, Sanitation, Hygiene and Waste Management for the COVID-19 Virus. Geneva, Switzerland: WHO.

[38] World Bank, ILO, WaterAid, WHO, 2019 Health, Safety and Dignity of Sanitation Workers. Washington, DC: World Bank.

[39] Bhowmick S, 2020 COVID-19: Indian healthcare workers need adequate PPE. BMJ Opin. Available at: https://blogs.bmj.com/bmj/2020/06/19/covid-19-indian-healthcare-workers-need-adequate-ppe/. Accessed July 1, 2020.

[40] Suryawanshi S, 2020 Community toilets main reason behind the rise of coronavirus cases in mumbai: BMC. The Indian Express. Available at: https://www.newindianexpress.com/cities/mumbai/2020/may/03/community-toilet-main-reason-behind-the-rise-of-coronavirus-cases-in-mumbai-bmc-2138756.html. Accessed September 7, 2020.

[41] Kuang J Ashraf S Das U Bicchieri C, 2020 Awareness, risk perception, and stress during the COVID-19 pandemic in communities of Tamil Nadu, India. PsyArXiv, 10.31234/osf.io/qhgrd. PMC7579253 33007992

[42] Caruso B 2019 Impacts of a Multi-Level Intervention, Sundara Grama, on Latrine Use and Safe Disposal of Child Faeces in Rural Odisha, India, 3ie Grantee Final Report. New Delhi, India: International Initiative for Impact Evaluation (3ie).

[43] Odagiri M Muhammad Z Cronin AA Gnilo ME Mardikanto AK Umam K Asamou YT, 2017 Enabling factors for sustaining open defecation-free communities in rural Indonesia: a cross-sectional study. Int J Environ Res Public Health 14: 1572. 10.3390/ijerph14121572 PMC5750990 29240667

[44] Government of India, 2018 Swachh Bharat Mission - Gramin, Ministry of Drinking Water and Sanitation. New Delhi, India: Government of India.

[45] Exum NG Gorin EM Sadhu G Khanna A Schwab KJ, 2020 Evaluating the declarations of open defecation free status under the Swachh Bharat (a € Clean India’) Mission: repeated cross-sectional surveys in Rajasthan, India. BMJ Glob Health 5: e002277.

[46] Luby SP Halder AK Tronchet C Akhter S Bhuiya A Johnston RB, 2009 Household characteristics associated with handwashing with soap in rural Bangladesh. Am J Trop Med Hyg 81: 882–887. 19861626 10.4269/ajtmh.2009.09-0031

[47] Biswas D Nizame FA Sanghvi T Roy S Luby SP Unicomb LE, 2017 Provision versus promotion to develop a handwashing station: the effect on desired handwashing behavior. BMC Public Health 17: 390. 28476170 10.1186/s12889-017-4316-6 PMC5420105

[48] Boland M Sweeney MR Scallan E Harrington M Staines A, 2006 Emerging advantages and drawbacks of telephone surveying in public health research in Ireland and the U.K. BMC Public Health 6: 208. 16911771 10.1186/1471-2458-6-208 PMC1560130

[49] Biran A Rabie T Schmidt W Juvekar S Hirve S Curtis V, 2008 Comparing the performance of indicators of hand-washing practices in rural Indian households. Trop Med Int Health 13: 278–285. 18304276 10.1111/j.1365-3156.2007.02001.x

